# Patchy fibrosis promotes trigger–substrate interactions that both generate and maintain atrial fibrillation

**DOI:** 10.1098/rsfs.2023.0041

**Published:** 2023-12-15

**Authors:** Michael A. Colman, Roshan Sharma, Oleg V. Aslanidi, Jichao Zhao

**Affiliations:** ^1^ School of Biomedical Sciences, University of Leeds, Leeds, UK; ^2^ Auckland Bioengineering Institute, University of Auckland, Auckland, New Zealand; ^3^ School of Biomedical Engineering and Imaging Sciences, King's College London, London, UK

**Keywords:** arrhythmia, atrial fibrillation, fibrosis, spontaneous triggers

## Abstract

Fibrosis has been mechanistically linked to arrhythmogenesis in multiple cardiovascular conditions, including atrial fibrillation (AF). Previous studies have demonstrated that fibrosis can create functional barriers to conduction which may promote excitation wavebreak and the generation of re-entry, while also acting to pin re-entrant excitation in stable rotors during AF. However, few studies have investigated the role of fibrosis in the generation of AF triggers in detail. We apply our in-house computational framework to study the impact of fibrosis on the generation of AF triggers and trigger–substrate interactions in two- and three-dimensional atrial tissue models. Our models include a reduced and efficient description of stochastic, spontaneous cellular triggers as well as a simple model of heterogeneous inter-cellular coupling. Our results demonstrate that fibrosis promotes the emergence of focal excitations, primarily through reducing the electrotonic load on individual fibre strands. This enables excitation to robustly initiate within these single strands before spreading to neighbouring strands and inducing a full tissue focal excitation. Enhanced conduction block can allow trigger–substrate interactions that result in the emergence of complex, re-entrant excitation patterns. This study provides new insight into the mechanisms by which fibrosis promotes the triggers and substrate necessary to induce and sustain arrhythmia.

## 1. Introduction

Fibrosis has been identified as a key component that drives dysfunction of the heart in multiple cardiovascular conditions [1,2] including atrial fibrillation (AF). Increased fibrosis can stiffen cardiac tissue, contributing to contractile dysfunction, and interrupt inter-cellular electrical coupling, contributing to electrical dysfunction, i.e. arrhythmia [3–6]. The contribution of fibrosis to arrhythmia may be particularly relevant for its role in AF. Characterized by rapid and irregular activation of the atria, AF is the most prevalent sustained cardiac arrhythmia that imposes a significant healthcare challenge in ageing societies [7,8]. Multiple studies have observed increased fibrosis in AF patients [9,10]. Fibrosis can lead to localized slowing of the excitation wavefront, functional conduction block, wavefront breakup, and potentially the generation of transient or sustained re-entrant circuits [4,11–13]; indeed, multiple studies have demonstrated that the specific patterns of arrhythmia can strongly depend on individual geometry and fibrosis distribution [6,14,15]. Thus, fibrosis has been comprehensively suggested to play a large part in the arrhythmia substrate associated with AF.

Whereas many studies have investigated the impact of fibrosis on the arrhythmia substrate, and more recently, on approaches for mapping fibrosis distributions and electrical excitation patterns [16,17], only a few have assessed its impact on arrhythmia triggers [18–20]. Triggers for arrhythmia can arise from various factors, including cellular spontaneous calcium release events (SCREs). SCRE can result in a whole-cell spontaneous calcium transient that activates the sodium–calcium exchanger (NCX), causing an inward current that can lead to a delayed after depolarization (DAD). If of sufficient magnitude, this DAD can cause the membrane potential to reach the activation threshold for the fast sodium current, *I*_Na_, and triggered activity (TA) may be observed. Previous studies have shown an important role of cellular connectivity in controlling the emergence of cellular SCRE as focal excitations in tissue [21–23]. Whereas the relationship between inter-cellular coupling and arrhythmia triggers is not entirely trivial, it is clear that reduced coupling (e.g. due to fibrosis) can help these independent cellular events overcome neighbouring electrotonic load and induce a full triggered excitation [18–20,23].

There are multiple presentations of fibrosis in the heart which may have differing implications for mechanical and electrical function [1]. For example, compact or replacement fibrosis involves regions of tissue where fibrosis has entirely replaced cardiomyocytes, substantially reducing contractility and leading to large permanent barriers to conduction. Patchy or interstitial fibrosis, commonly observed in AF patients, involves the separation of myocardial bundles which can increase local conduction heterogeneity and anisotropy [24,25]. This may be conducive to arrhythmia generation through the formation of temporary, functional barriers to conduction. Such an underlying conduction substrate could promote breakup of the excitation wavefront which may lead to self-sustaining re-entry, as has been indicated in previous studies (e.g. [4]). Moreover, the transformation of the underlying tissue structure from a continuous syncytium to a loosely connected collection of one-dimensional strands or bundles [18] may have significant implications on the electrotonic load interactions that control the emergence of focal excitations and the subsequent evolution of the excitation pattern to form a sustained arrhythmia.

This study aims to explore multiple factors that underlie the generation of arrhythmia in the presence of fibrosis. We will elucidate the mechanisms that underlie arrhythmogenesis, with a focus on DAD-mediated triggers and their potential interaction with the arrhythmia substrate. To achieve this, we combine our reduced model of stochastic cellular SCRE [23] with our recently presented model of heterogeneous inter-cellular coupling [26]. These models enable feasible simulations to be performed while fully controlling for the inter-cellular coupling condition and SCRE dynamics, and therefore facilitate rigorous correlation between these features and emergent behaviour.

## 2. Methods

### 2.1. Models of cellular electrophysiology

**Figure 1.**
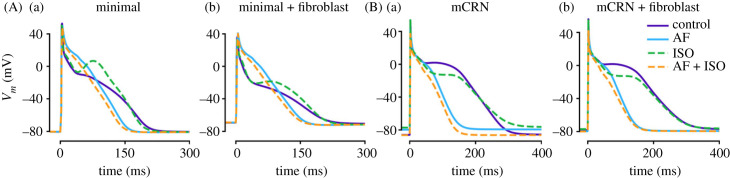
Single cell action potential models of the human atrial myocyte. Action potentials produced by the cellular models used in this study at a cycle length of 1000 ms are shown for the minimal model (A) and (mCRN) [27] model (B) for a range of conditions: control and AF (solid lines), and ISO and ISO + AF (dotted lines), without (a) and with (b) fibroblast coupling.

All simulations were performed using our previously presented framework in C++ [23]. This framework integrates detailed spatial cellular models, reduced models of SCRE, traditional non-spatial cellular models and tissue models into a consistent package. For use in this study, the reduced models of SCRE are required to efficiently simulate spontaneous activity and therefore all cell models must be integrated with the structure of the intracellular calcium handling system presented in that model. First, we implement the minimal model which was designed to have as few components as possible while still being suitable for integration with biophysically detailed models of the calcium currents that are coupled to the intracellular calcium handling system. The model can be parameterized to represent the electrophysiology of the atrial working myocardium (figure 1Aa).

To investigate dynamics under a variety of conditions, simple models of AF remodelling and sympathetic stimulation (ISO) were also included (see electronic supplementary material, S1—Model description for further details). AF remodelling was included primarily for investigation of the arrhythmia substrate, where inducibility of sustained re-entry is an important factor to quantify. ISO was used to help maintain high loads of the sarcoplasmic reticulum (SR) calcium concentration, [Ca^2+^]_SR_, especially in the context of AF remodelling (which reduces the [Ca^2+^]_SR_ and magnitude of the intracellular calcium transient compared to control). This was primarily implemented in investigation of [Ca^2+^]_SR_ dependence or to study the interaction between re-entry and triggers, such that [Ca^2+^]_SR_ load can be maintained even during rapid pacing (see Methods—Simulation protocols, below). Simulations were performed across this parameter spectrum to ensure all behaviour was captured. In order not to convolute the story of results, those selected for visualization and presentation were primarily in control and non-ISO conditions, except in cases where the introduction of remodelling or ISO had important impacts on dynamics; in these cases, this will be clearly stated.

Furthermore, modelling studies have previously shown that electrical coupling between fibroblasts (a component of fibrosis) and cardiomyocytes can act to raise the resting potential of the latter [19,28–32]. Resting potential is an important determinant of TA, governing both the size of DAD required to reach the *I*_Na_ threshold and the degree of inactivation of *I*_Na_ when this threshold is met. Therefore, we also include a simplified model of myocyte–fibroblast coupling that reproduces this impact on cellular resting membrane potential (figure 1Ab), but without the biophysical detail of coupling myocytes to excitable fibroblasts that has been included in other studies (e.g. [4,16,28]).

To supplement these simulations, we also implemented a simplified and modified version of the Courtemanche *et al.* [27] human atrial cell model (mCRN): the main ion currents were extracted from the original model and merged with the calcium handling system of our model, with some parameters adjusted accordingly. AF remodelling, ISO and fibroblast coupling were implemented into this model in the same way as the minimal model (figure 1B). This model was used to verify observations in the minimal model. Results from this model are generally not presented, except for in cases where it resulted in different behaviour to the minimal model. Please see electronic supplementary material, S1—Model description for further details of model construction.

### 2.2. Reduced model of spontaneous calcium release and delayed after depolarizations

**Figure 2.**
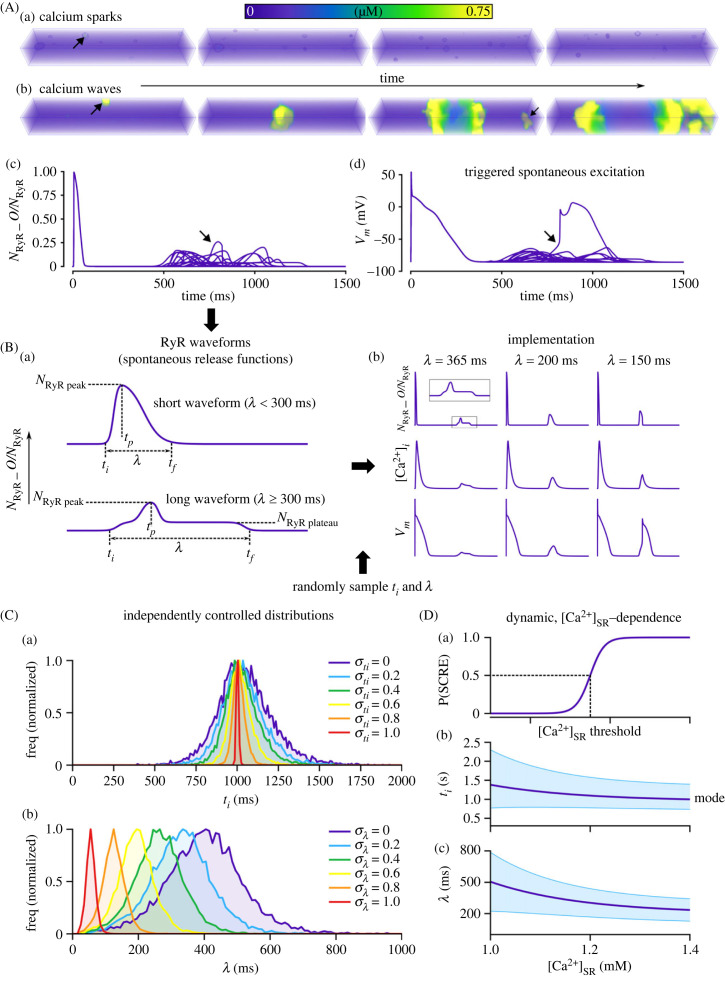
Reduced model of spontaneous calcium release. (A) Illustration of calcium sparks (a) and waves (b), and the whole-cell RyR waveform (c) and voltage deflection that results (d). (B) Illustration of the RyR waveform with labelled parameters (a) and the impact of implementing waveforms with different parameters into the cell model (b). (C) Illustration of the independently controlled distributions from which waveform parameters are randomly sampled, showing the distributions at varying values of the input control parameters, *σ*_ti_ and *σ_λ_*. (D) Dependence of distribution parameters on [Ca^2+^]_SR_ for dynamic simulations.

A reduced and computationally efficient model that captures the underlying stochasticity and variability of SCRE is required to feasibly perform tissue simulations. We implement our previously presented approach [23,33] which involves the use of fully controllable spontaneous release functions (SRFs). It is worth first considering the behaviours that the reduced models aim to capture. Spontaneous calcium sparks (figure 2Aa) may manifest as whole-cell calcium waves or multiple wavelets (figure 2Ab). This is underlain by a transient opening of the ryanodine receptors (RyR; figure 2Ac), the channels responsible for intracellular calcium release, which results in a spontaneous calcium transient that can activate NCX and lead to DADs or TA (figure 2Ad). It is the range of waveforms of this transient RyR activity that the reduced model aims to capture. By defining simple analytical functions (the SRF) that describe the underlying RyR waveforms (figure 2Ba), it is possible to impose SCRE in non-spatial cell models (figure 2Bb). For a simple, spike-like morphology, the waveform is given by [23]

2.1 $$N_{\mathrm{RyR}\_O}=N_{\mathrm{RyR}\_O}^{\mathrm{peak}}{\left[\left(1+e^{-(t-t_{1})/k_{1}})(1+e^{-(t-t_{2})/k_{2}}\right)\right]}^{-1},$$

2.2 $$t_{1}=t_{i}+0.5(t_{p}-t_{i}),$$

2.3 $$t_{2}=t_{p}+0.5(t_{f}-t_{p}),$$

2.4 $$k_{1}=0.1689(t_{p}-t_{i})+0.00255,$$

2.5 $$\mathrm{and}\;k_{2}=0.1689(t_{f}-t_{p})+0.00255,$$

where *t_i_* is the initiation time of the SCRE, *t_f_* is the end time (duration, *λ*, thus = *t_f__−_ t_i_*), *t_p_* is the time of the peak of the waveform and *N*_RyR_O_^peak^ is the peak of open proportion RyR. The function for the plateau-like waveform (corresponding to durations longer than 300 ms) is defined similarly

2.6 $$N_{\mathrm{RyR}\_O}=\begin{array}{c} N_{{\;}_{\mathrm{RyR}\_O}}^{\mathrm{plateau}}{\left[\left(1+e^{-(t-(t_{i}+17.5))/5.946})(1+e^{(t-(t_{f}-17.5))/5.946}\right)\right]}^{-1}+\\ (N_{{\;}_{\mathrm{RyR}\_O}}^{\mathrm{peak}}-N_{{\;}_{\mathrm{RyR}\_O}}^{\mathrm{plateau}}){\left[\left(1+e^{-(t-(t_{p}-25))/5.946})(1+e^{(t-(t_{p}+17.5))/5.946}\right)\right]}^{-1} \end{array},$$

where *N*_RyR_O_^plateau^ is the amplitude of the plateau. The waveform is, therefore, completely described by four or five parameters: (i) initiation time, *t_i_*; (ii) duration, *λ* *=* *t_f__−_ t_i_*; (iii) peak time, *t*_p_ and (iv,v) amplitude (*N*_RyR_O_^peak^; *N*_RyR_O_^plateau^). To maintain simplicity in controllability, the peak time can be defined from *λ* and *t_i_*, and the amplitudes (*N*_RyR_O_^peak^; *N*_RyR_O_^plateau^) can both be defined by the *λ*. Therefore, the model is reduced to take two input parameters from which everything else is defined: *λ* and *t_i_*.

By randomly sampling these two parameters, it is possible to capture the underlying stochasticity and variability of SCRE in a fully controllable manner. The distributions from which these parameters are sampled can be varied independently of each other and are typically non-normal (electronic supplementary material, S1—Model description section, S3.2). Sigmoidal functions are used to describe the corresponding cumulative frequencies, such that a random number passed into the inverse function will return the parameter value with the appropriate probability. For example, for *t*_i_, the sigmoid describing the cumulative frequency is given by

2.7 $$F(t_{i})=\begin{array}{c} F_{1}(t_{i})=(2{\mathrm{CF}}_{t_{i,\mathrm{Sep}}}){\left(1+e^{-\left(t_{i}-t_{i,\mathrm{Sep}}\right)/k_{F_{1}}}\right)}^{-1}\\ F_{2}(t_{i})=(2(1-{\mathrm{CF}}_{t_{i,\mathrm{Sep}}})){\left(1+e^{-\left(t_{i}-t_{i,\mathrm{Sep}}\right)/k_{F_{2}}}\right)}^{-1}-1+2{\mathrm{CF}}_{t_{i,\mathrm{Sep}}} \end{array}\}\begin{array}{c} t_{i}<t_{i,\mathrm{Sep}}\\ t_{i}\geq t_{i,\mathrm{Sep}} \end{array},$$

where *t_i_*__sep_ and the independent gradient parameters (*k_F_*_1_ and *k_F_*_2_) are used to account for the skewness of the distribution. The function is defined by the value of *t_i_*__sep_, the cumulative frequency at this value (CF_ti_sep_), and the gradient parameters for each region. The inverse function, to return a *t_i_* from a given random input, is

2.8 $$t_{i}=\begin{array}{c} -k_{F_{1}}.\ln\left(\frac{2{\mathrm{CF}}_{t_{i,\mathrm{Sep}}}}{\mathrm{rand}}-1\right)+t_{i\_\mathrm{sep}}\\ -k_{F_{2}}.\ln\left(\frac{2(1-{\mathrm{CF}}_{t_{i,\mathrm{Sep}}})}{\mathrm{rand}+1-2{\mathrm{CF}}_{t_{i,\mathrm{Sep}}}}-1\right)+t_{i\_\mathrm{sep}} \end{array}\}\begin{array}{c} \begin{array}{c} \mathrm{rand}<{\mathrm{CF}}_{t_{i,\mathrm{Sep}}}\;\\ \; \end{array}\\ \mathrm{rand}\geq {\mathrm{CF}}_{t_{i,\mathrm{Sep}}}. \end{array}$$

The same process is followed for *λ* (electronic supplementary material, S1—Model description section, S3.2 and S3.5), enabling the values of both parameters to be randomly sampled with appropriate, defined probabilities.

To simplify for analysis and interpretation purposes, we introduce two parameters to control these distributions independently (figure 2C): the *t_i_* control variable, *σ*_ti_, and the *λ* control variable, *σ_λ_*. Both vary from 0 (widest distributions and longest median duration) to 1.0 (tightest distributions and shortest median duration). Simple linear equations were used to relate the input parameters (*σ*_ti_ and *σ_λ_*) to the SRF distribution parameters (electronic supplementary material, S1—Model description section, S3.3.1 and table S9). The probability of SCRE was set to 1.0 for all of these conditions.

Alternatively, the distributions can be defined as a function of [Ca^2+^]_SR_ to enable the model to respond dynamically to environmental variables (figure 2D). Here, all parameters are nonlinear functions of [Ca^2+^]_SR_ (electronic supplementary material, S1—Model description section, S3.4, figure S2 and table S10). In these conditions, the probability of SCRE transitions from 0.0 to 1.0 around a defined [Ca^2+^]_SR_ threshold (figure 2Da).

In all simulations, SCRE distributions are homogeneous across the whole tissue, i.e. whereas individual nodes will have their own randomly sampled parameters to describe SCRE, these parameters are all sampled from the same distributions throughout the tissue; the distributions themselves are the control variable against which observations are related. Note that this does not mean every node will necessarily undergo SCRE within the time of the simulation, as the sampled initiation time could be beyond the simulation time. Simulations in the [Ca^2+^]_SR_ threshold region will also contain conditions where the probability of cellular SCRE is below 1.0, in which case, only a proportion of the nodes will actually undergo SCRE (although each node has an equal probability of doing so).

### 2.3. Tissue and fibrosis models

**Figure 3.**
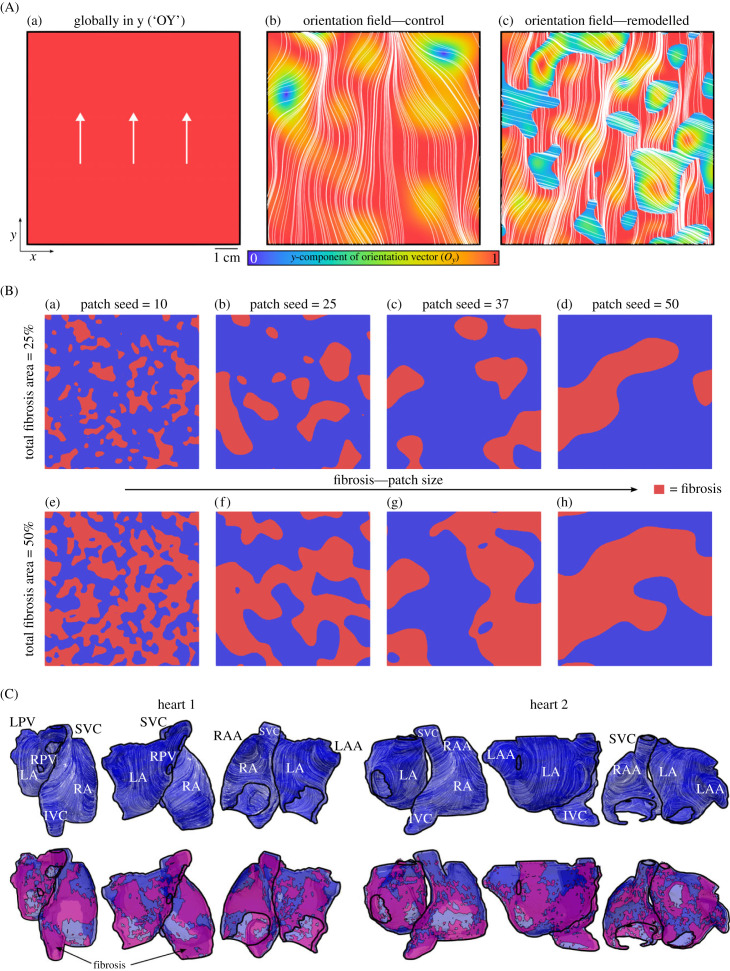
Tissue models. (A) Two-dimensional tissue models used in this study, showing the myocyte orientation in the three different models corresponding to the model where all fibres point in *y* (a, referred to as ‘OY’ for brevity in the manuscript), and the vector field models representing control (b) and remodelled (c) fibre structure. The colour scale corresponds to the magnitude of the *y*-component of the orientation vector, and streamlines are shown to illustrate fibre structure itself. (B) Various fibrosis distributions used in the two-dimensional tissue models, corresponding to different patch size seeds (left to right) and different total area of fibrosis (upper and lower). (C) Three-dimensional, anatomically detailed tissue models implemented in this study, showing the two atria from three different views. Myocyte orientation is illustrated with white streamlines on the upper panels, with various regions labelled for context and clarity (note that the electrophysiology was homogeneous in simulations in this study); lower panels show patient specific fibrosis distribution in pink.

The majority of simulations were performed in idealized two-dimensional tissue models, which readily facilitate full control over the various parameters of tissue structure and enable correlation between observed dynamics and underlying microstructure. Three baseline two-dimensional tissue models were implemented in this study, with different structures of myocyte fibre orientation. The first model is fully idealized and contains myocyte orientation globally pointing in the *y*-direction, referred to as ‘OY’ (figure 3Aa). The other two models employ spatially varying orientation fields corresponding to a ‘control’ model, which contains smooth changes to orientation (figure 3Ab), and a ‘remodelled’ model, which contains abrupt changes to myocyte orientation, representing fibre disorganization associated with AF (figure 3Ac). The specific fibre structures of these tissue models were not considered in detail and were not directly validated; the purpose of these different models was to explore whether fundamentally different types of structure (fully ideal, smoothly varying, abrupt changes) would impact dynamics in a relevant way.

Patchy fibrosis was implemented into these two-dimensional tissue models to describe a variety of conditions regarding patch size and total area occupied by fibrosis. Gaussian random fields were used to generate spatially varying patterns (e.g. [12,34]), and thresholds were then applied to impose a total fibrotic area of either 25% or 50% of the tissue. This allowed small (seed = 10) and large (seed = 50) fibrosis patches to be imposed at each desired total fibrosis area (figure 3B).

Two three-dimensional late gadolinium enhanced (LGE)-MRI clinical data were sourced from patients with AF at Waikato Hospital, Waikato, New Zealand, between June 2017 and February 2020 (Health and Disability Ethics Committee, Ref: 16/STH/130). The three-dimensional computer models incorporated LGE-MRI-derived atrial anatomies, fibrosis distributions and three-dimensional rule-based fibre orientations (figure 3C). The rule-based fibres were generated through a semi-automatic approach that used a weighted distance function of orientation indicating vectors, as discussed in the original study from which these geometries were provided [35].

**Figure 4.**
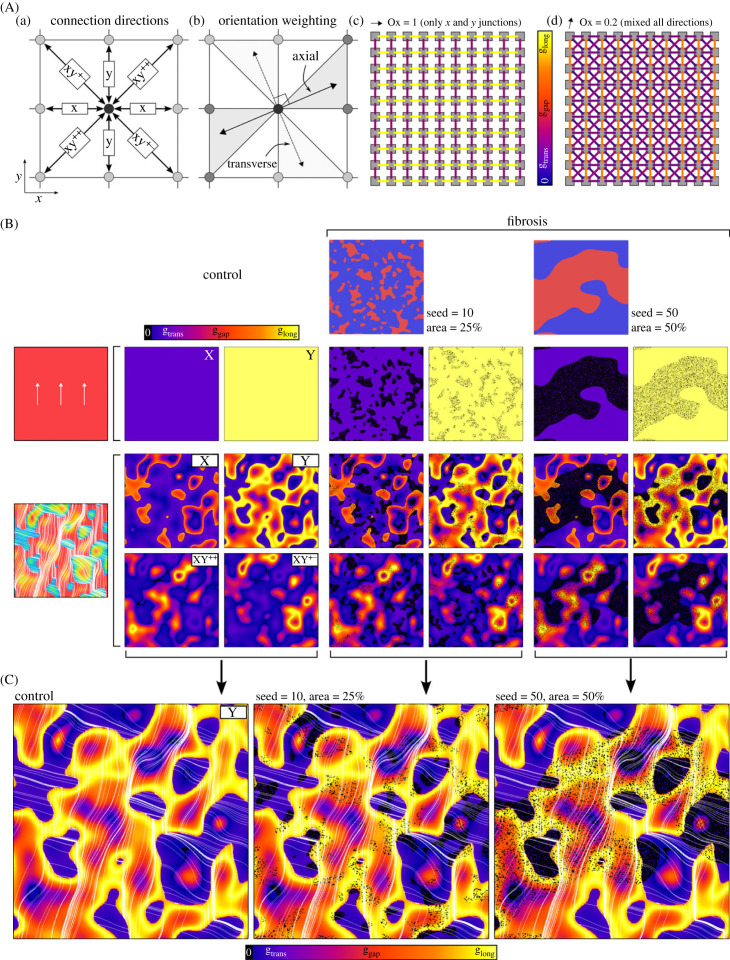
Models of fibrosis. (A) Illustration of the network model of inter-cellular coupling implemented in this study, showing the connection directions (a), orientation weighting (b) and the magnitude of the network connections in two idealized fibre conditions (c and d). Here, individual nodes and connections between nodes in all directions are shown on a single panel. (B) Connection maps illustrated for control and two different fibrosis distributions in the OY and remodelled fibre orientation models. Here, the magnitude of connection is shown for each direction on its own panel due to the larger tissue size. For the OY condition, connections are only non-zero in the *x*- and *y*-directions and so only these are shown; for the remodelled fibre orientation condition, connections are present for all four coupling directions and are all shown explicitly. Colours represent the strength of the connection between two adjacent nodes at each location and in each direction; the pattern is determined by both local orientation and fibrosis distribution. (C) Zoomed view of the connection maps and relation to myocyte orientation in control and the two fibrosis models, showing only the map in the *y*-direction. Orientation streamlines are shown to help relate coupling strength to both fibrosis and orientation.

Our recently proposed network model of inter-cellular coupling was implemented in the present study due to its ability to easily and directly control inter-cellular connections, differentially in the longitudinal and transverse directions [26]. The reader is referred to the original study and electronic supplementary material, S2 for full details on the method, which will be only briefly described here. The model is most clearly illustrated in the two-dimensional models. Nodes can be connected to up to eight other neighbouring nodes (in a regular, structured-grid geometry), representing four independent directions (figure 4Aa). The direction of the myocyte orientation vector is used to generate weighted axial and transverse connections between neighbouring nodes (figure 4Ab). Connection maps in simple cases where the orientation points globally either in axis or between axes are illustrated (figure 4Ac). Note that only two directions are non-zero in the case where the orientation points exactly along an axis or diagonal, whereas all four directions are non-zero when the orientation is not exactly along an axis or diagonal. Within fibrosis patches, 80% of transverse connections and 20% of longitudinal connections were randomly removed (figure 4B) to represent the separation of myofibre bundles [1,24,25,36]. Inter-cellular connections, therefore, depend on both local myocyte orientation and fibrosis-condition (figure 4C).

All two-dimensional tissue models are 300 × 300 nodes large with an inter-nodal spacing, Δ*x*, of 0.3125 mm (to match the three-dimensional models). All simulations were performed with an integration time step, Δ*t*, of 0.01 ms, which was demonstrated to be comfortably within the range for stability and model convergence while also enabling practical simulation times at the spatial step implemented. Inter-cellular coupling strength in the axial direction, *g_a_*, was 1.28 nS/pF and the anisotropy ratio was set to 7 : 1, giving a transverse coupling strength, *g_t_*, of 0.183 nS/pF, resulting in conduction velocities of 1.27 and 0.4 mm ms^−1^ and an overall activation time in control, anatomically detailed atria of approximately 130 ms [37].

### 2.4. Simulation protocols and conditions

Single cell models were pre-paced at multiple cycle lengths until they reached steady-state, at which point the state variables were saved. Multiple cycle lengths were used because different aspects of our analysis and protocols required different pacing rates. The majority of simulations used a cycle length of 400 ms. This relatively fast pacing rate was used as it ensured a reasonably large [Ca^2+^]_SR_ load during steady-state, which is important for SCRE magnitude. However, in investigations of the arrhythmia substrate, faster pacing rates were used (corresponding to cycle lengths of 150, 200 and 250 ms); in investigations of the interaction between sinus pacing and spontaneous triggers, a slower pacing rate was implemented that corresponds to sinus rhythm (cycle length = 1000 ms) and provides time for SCRE to occur between paced beats. Stimuli were applied by injecting a current of magnitude −12.5 pA/pF for five ms at regular intervals defined by the cycle length. In two dimensions, stimuli were either applied to the centre of the tissue (most simulations) or to the inferior edge (rapid pacing for substrate simulations). In three-dimensional models, stimuli were applied to a region of the right atrium near the superior vena cava, approximately correlating with the location of the sino-atrial node (SAN). Tissue models were paced for five beats, initially using the saved state variables from the single cell models, to enable the tissue rapidly to reach a steady-state.

Simulations were performed by reading in the steady-state tissue variables, applying one stimulus and letting the model evolve following the application of the SRF. Some simulations involved applying multiple stimuli, which will be clarified in the relevant results section. Given that the steady-state [Ca^2+^]_SR_ differs between each model and condition, it is important to control for this variable due to the impact that this has on the magnitude of calcium release for a given SRF profile (the magnitude of calcium release for a given *N*_RyR_open_ profile is positively correlated with [Ca^2+^]_SR_). To avoid this heterogeneous impact and ensure that behaviour can be confidently related to the SRF parameters themselves, the [Ca^2+^]_SR_ was set to exactly 1.0 mM after refilling from the first paced beat but before any SCRE were initiated. In simulations directly relating [Ca^2+^]_SR_ to the onset of focal excitations, the corresponding [Ca^2+^]_SR_ was set to exactly the value desired, rather than 1.0 mM, in the same way.

In simulations focusing on the emergence of focal triggers, the probability of triggered activity was given as the number of simulations in which a spontaneous focal beat occurred as a proportion of the total number of simulations for each condition (that is, each individual combination of cell model, tissue model, and SRF control variables or [Ca^2+^]_SR_ input) To make this a computationally tractable study, a strategy was implemented in which the first pass over all conditions was just a single simulation. This was used to identify the threshold region for each condition. Then, a further 29 simulations were performed (giving 30 overall) in a broad parameter range that fully encapsulated the threshold region for determining the probability of focal excitation for each condition. The priority was to ensure that the entire region where the probability transitioned from zero to one was captured, and therefore many simulations at the edges of this region were essentially redundant (all 30 giving either one behaviour or the other). Despite this strategy, the number of simulation hours required to perform such a study is still exceptionally high. Simulations were, therefore, performed using ARC3 and ARC4, part of the High Performance Computing facilities at the University of Leeds, UK, and would not have been feasible without access to these facilities.

## 3. Results

### 3.1. Effect of patchy fibrosis on activation time and activation wavefronts

**Figure 5.**
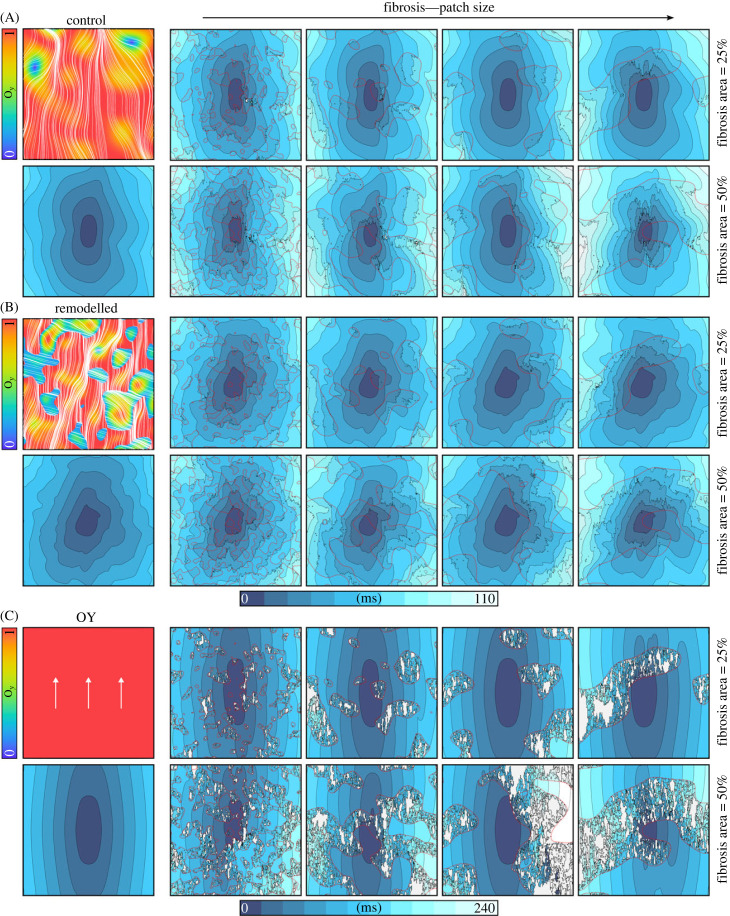
Activation maps in the two-dimensional tissue models. Activation maps are shown for all of the fibrosis models in the three two-dimensional geometries (A–C). The orientation field is shown for completeness above the control activation pattern. The colourmap for activation times is the same for (A) and (B), illustrated below (B), with (C) having its own colourmap. Results shown are for the minimal cell model in control conditions without ISO, and a pre-pacing cycle length of 400 ms.

The presence of patchy fibrosis had notable effects on the overall activation time and activation wavefront patterns (figure 5). The regions with fibrotic tissue exhibited a slowing down of conduction, particularly when the wavefront was perpendicular to the myocyte orientation, manifesting as areas of conduction delay. This resulted in more heterogeneous activation wavefronts across the tissue. Overall activation time and conduction velocity in the direction parallel to the fibre structure was largely unchanged in the models, whereas in the transverse direction it was markedly reduced, by a factor of up to approximately two.

Fibre orientation remodelling had distinct effects on the conduction properties (figure 5B). In the remodelled condition, abrupt changes to the fibre orientation were observed as well as a more substantial representation of orientation pointing in the *x*-direction (transverse to the global average of *y*). This led to more spatially heterogeneous patterns of conduction, but an overall reduction in the activation time due to the increased lateral conduction.

In the fully idealized model (OY), where all fibres were entirely oriented in the *y*-direction, the effects of fibrosis were significantly emphasized and exaggerated (figure 5C). This idealized arrangement of fibres oriented in one direction led to substantial conduction block in the transverse (*x*) direction when fibrotic regions were present. This contributed to substantial conduction delay, and also led to the failure of excitation to reach some regions of tissue where no robust connections to neighbouring tissue were present. The same features were observed across all model conditions investigated (i.e. with and without remodelling or ISO and in both the minimal and mCRN cell models).

### 3.2. Fibrosis promotes the emergence of focal excitations

**Figure 6.**
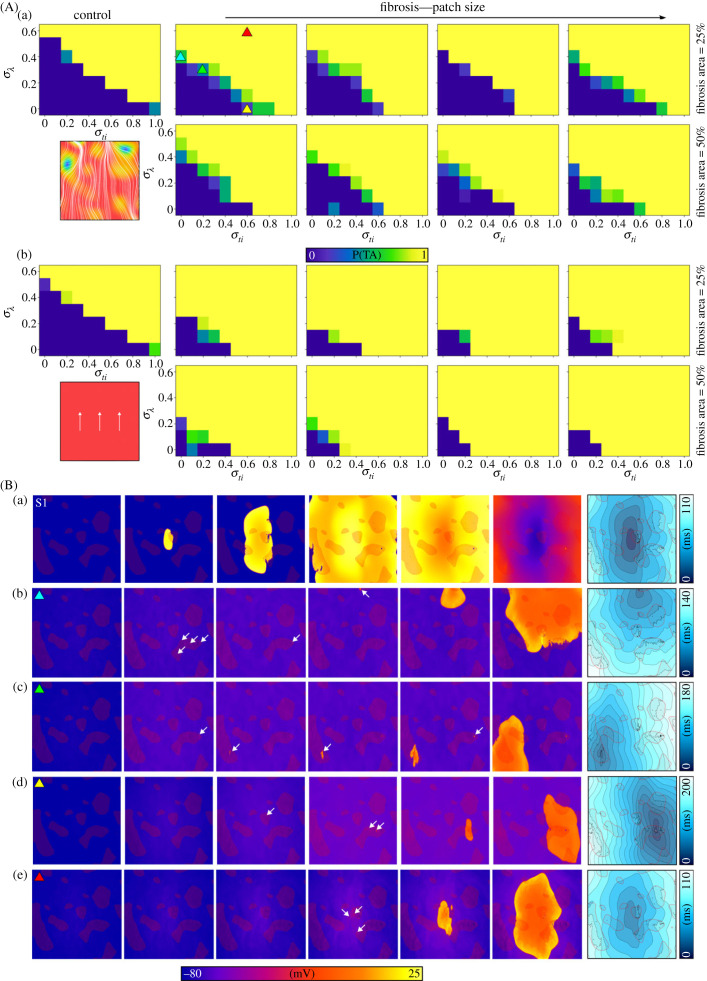
Impact of fibrosis on the conditions required for a focal excitation. (A) Probability of a focal excitation (P(TA)) at different combinations of the distribution control parameters (*σ*_ti_ and *σ_λ_*) for all fibrosis models in the control (a) and OY (b) tissue geometries. Colourmap indicates P(TA). (B) Example activation patterns for the paced beat (a) and four different focal excitations (b–e), corresponding to different regions of the parameter space; temporal voltage snapshots and activation map are shown. These regions are indicated in A by the coloured triangular markers. White arrows indicate regions of large cellular TA that may or may not fully induce a focal excitation, and fibrosis patches are shown in semi-transparent dark red for context and relation of spatial properties. Results shown are for the minimal cell model in control conditions without ISO, and a pre-pacing cycle length of 400 ms.

Across all fibrosis models, the requirements on the duration and timing of SCRE to induce a focal excitation were less constrained compared to the control scenario (figure 6A). No clear pattern correlating the size of the fibrotic patches with focal excitation vulnerability was observed; rather, the specific pattern and relation to activation pattern may be more important. However, the overall percentage of fibrosis within the tissue had a marked impact on the probability of focal excitations: higher fibrosis percentages were associated with an increased likelihood of focal excitations occurring.

The presence of fibrosis also induced more substantial probabilistic regions compared to the control scenario. In control, the emergence of focal excitations was typically characterized by an ‘all-or-nothing’ response. By contrast, the introduction of fibrosis led to a broader spectrum of probabilities for focal excitations near the threshold region. However, this threshold region was still narrow in all simulations. The idealized OY model once again exaggerated the impact of fibrosis compared to the spatially varying fields (figure 6Ab versus Ba). This aligns with the proposed mechanism by which fibrosis promotes arrhythmia triggers (next section).

**Figure 7.**
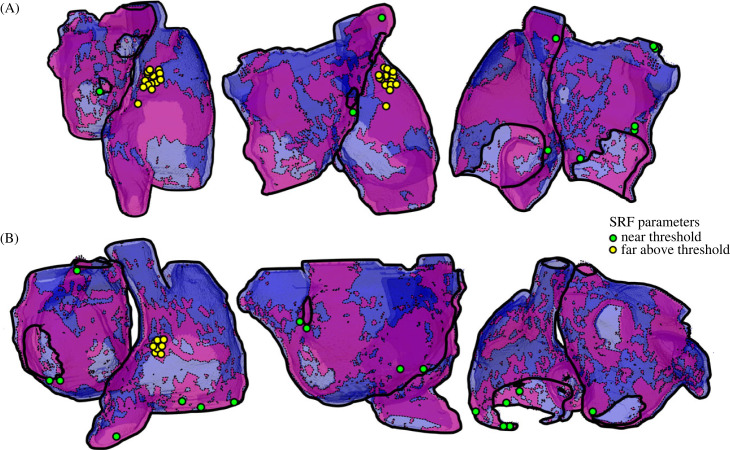
Focal locations in three-dimensional anatomically detailed atrial models. Indications of the location of focal sources across heart 1 (A) and heart 2 (B) from three different views (the same as those in figure 3). Each circle marker corresponds to the locus of a spontaneous focal excitation. Markers in green are from simulations with SRF parameters that are close to the threshold for focal excitation; yellow markers indicate simulations wherein the SRF parameters were far above the threshold. Fibrosis is shown as pink regions with dotted outlines.

The focal patterns that emerged at either end of the SCRE threshold region exhibited similar characteristics (figure 6B, electronic supplementary material, video S1). For scenarios with the loosest constraints on timing and tightest on duration, or vice versa, a single focus emerged from within a fibrotic patch. Additionally, we observed that edge effects were significant in influencing focal excitation patterns. Under parameter combinations that did not induce focal excitations in the control model, excitations in fibrosis simulations were always localized to fibrotic patches. When SCRE parameters were significantly above the excitation thresholds, the focus location was consistently found to be close to the original stimulus location. This proximity can be attributed to the increased waiting time at the stimulus site due to its earliest activation: this region undergoes SCRE at the earliest time, and if this is sufficient to induce a focal excitation, it will do so from here. While the focal location still showed a relation to the fibrosis pattern, it was not entirely localized to these patches, and focal excitations were also observed without fibrosis under these parameter combinations. These results were reflected in the three-dimensional anatomically detailed atrial models, where focal excitations were preferentially located to regions of fibrosis (figure 7). Close to the threshold, focal locations were observed across many regions of the right and left atria; far above this threshold, focal locations were primarily observed proximal to the original pacing site. Edge effects were also important near the threshold regions: here, focal excitations were preferentially located near edges of the tissue such as proximal to the valves or openings of the veins.

### 3.3. Loss of transverse connections is the primary mechanism that promotes focal excitations

**Figure 8.**
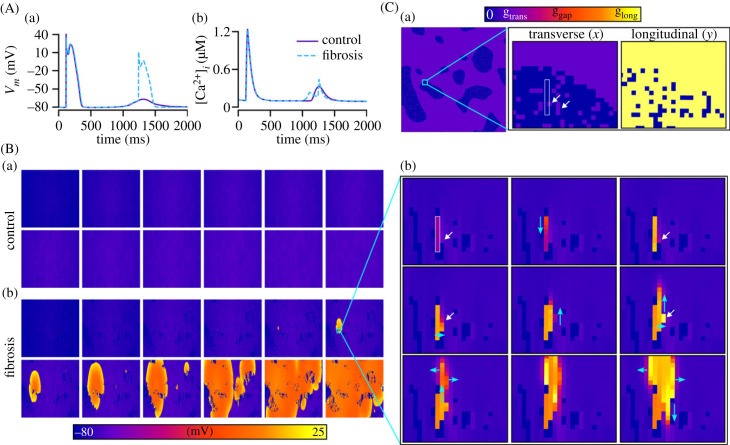
Illustration of the mechanism of induction of a focal excitation. (A) Voltage and calcium traces from a simulation in control and fibrosis that have the same spontaneous calcium release parameters. (B) Voltage snapshots for both simulations, illustrating DADs that do not induce a focal excitation in control (a) and the induction of a focal excitation in fibrosis (b). (C) Zoomed view to illustrate the mechanism. Connection maps are shown for the zoomed region for context (a), with a small one-dimensional strand highlighted. White arrows point to lateral connections that are relevant for the excitation pattern. Panel (b) shows temporal snapshots of this zoomed region. Blue arrows show direction of wavefront propagation while white arrows with the triangular arrowhead indicate important features/regions. Results shown are for the minimal cell model in control conditions without ISO, and a pre-pacing cycle length of 400 ms.

The mechanism by which fibrosis promotes arrhythmia triggers is most clearly illustrated in the idealized OY tissue model (figure 8, electronic supplementary material, video S2), where individual fibre ‘strands’ can be easily visualized (all connections in *y* are along a strand and all connections in *x* are transverse to it). The ectopic focal excitation originates from a small region of tissue before spreading throughout the whole tissue. By zooming in on this local region, it is clear that excitation was initiated from a short strand that contained very few transverse connections (figure 8C). These regions, characterized by a scarcity of cross-fibre connections, provide an environment where DADs more easily manifest as TA [18,19]. Consequently, the TA propagates along the almost isolated one-dimensional strand formed by the fibrotic patch before eventually reaching transverse connections and ‘jumping’ to the adjacent strand. In this case, the excitation then travels upwards along this second strand, before jumping over to a third strand and eventually propagating into the remaining tissue sufficiently to induce a full focal excitation.

### 3.4. Fibrosis promotes arrhythmia substrate through heterogeneously slowed conduction

**Figure 9.**
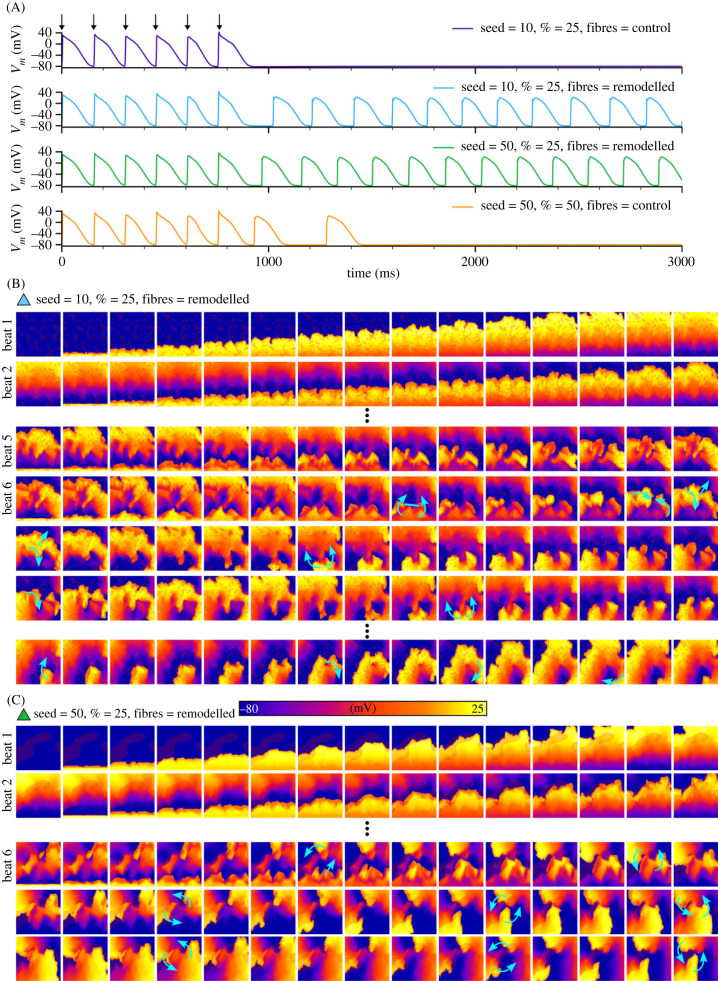
Rapid pacing leads to re-entry. Illustration of different behaviours following rapid pacing, showing action potential traces (A) and voltage snapshots (B,C) in different conditions. Blue arrows indicate the direction of wavefront propagation and blue lines represent regions of conduction block, and fibrosis patches are shown in semi-transparent dark red for context and relation of spatial properties. Vertical ellipses indicate a jump in time between rows of snapshots. Fibrosis is shown in the dark, semi-transparent red and is most easily seen in the first snapshot of each panel. Snapshots in (B) correspond to the fibrosis case of patch seed = 10 and total area = 25%; (C) corresponds to seed = 50 and area = 25%, as indicated by the colour of the triangular marker that corresponds to the colour of the traces in (A). It may be clearer to relate the voltage patterns observed to the fibrosis patterns as shown in figure 3B and in electronic supplementary material, video S3. Results shown are for the minimal cell model with AF remodelling and without ISO, and a pacing cycle length of 150 ms.

Numerous previous studies have provided evidence that fibrosis promotes the formation of re-entrant circuits that drive arrhythmia (e.g. [4,11,12]). Because these findings are well-established, we will not perform extensive simulations, but rather will simply demonstrate these dynamics in our model (figure 9). By applying rapid pacing (five beats at a cycle length of 150 ms) in the context of AF remodelling, it was possible to induce re-entry in the fibrosis models (figure 9). Breakup of the excitation wavefront could degenerate into transient or sustained re-entry with multiple emergent patterns: (i) no re-entry, (ii) transient re-entry that self-terminates, (iii) sustained re-entry that dynamically evolves before eventually settling into a stable pattern (figure 9B) and (iv) sustained re-entry that quickly finds a stable driver (figure 9C). Different re-entrant patterns were observed, including single scroll waves, figure-of-eight waves (two scroll waves rotating in opposite directions), and dual rotating waves that repeatedly detach and re-attach (figure 9C, electronic supplementary material, video S3). In all cases, the conduction patterns were highly correlated with fibrosis structure. Fibre structure remodelling also promoted the generation of sufficient conduction block that could lead to re-entry.

### 3.5. Fibrosis-induced focal excitations can interrupt normal pacing

**Figure 10.**
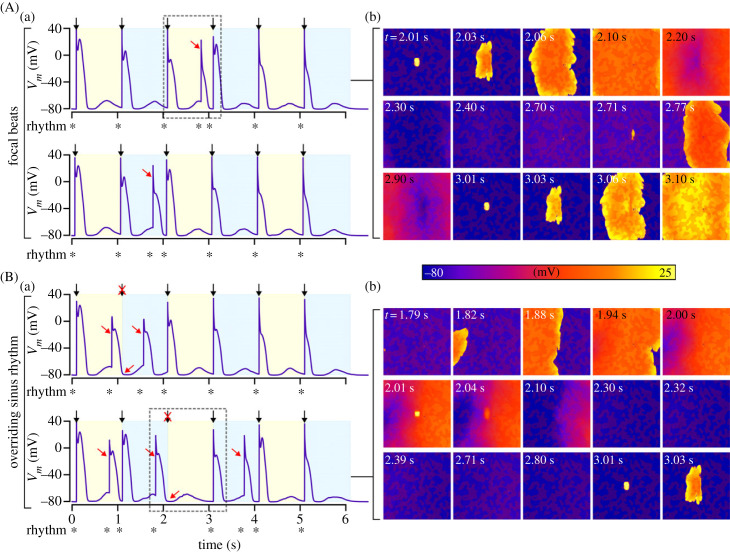
Interruption of sinus rhythm. Various simulations in which sinus rhythm was interrupted are shown, for two different behaviours of focal beats occurring between sinus pulses (A) and focal beats that can override sinus pulses (B), showing action potential traces (a) and voltage snapshots (b). Black arrows indicate applied stimuli (also shown by the alternating shading) and red arrows point to relevant features. Fibrosis patches are shown in semi-transparent dark red for context and relation of spatial properties. The rhythm (activation intervals) is shown below each panel, indicated by *. Results shown are for the minimal cell model in control conditions without ISO, and a pacing cycle length of 1000 ms.

Our investigation revealed that fibrosis can lead to focal excitations occurring occasionally within normal sinus rhythm (figure 10, electronic supplementary material, video S4; cycle length = 1000 ms). Depending on the SRF parameter distributions, focal excitations may occur between sinus beats, either as isolated occurrences or between multiple consecutive beats (figure 10A). The timing and pattern of these focal excitations can also lead to the suppression of the sinus beat (figure 10B). Both forms of interruption resulted in abnormal rhythms characterized by variability in the excitation interval, with both shorter and longer intervals observed.

### 3.6. Fibroblast coupling provides the substrate for spontaneous generation of arrhythmia

**Figure 11.**
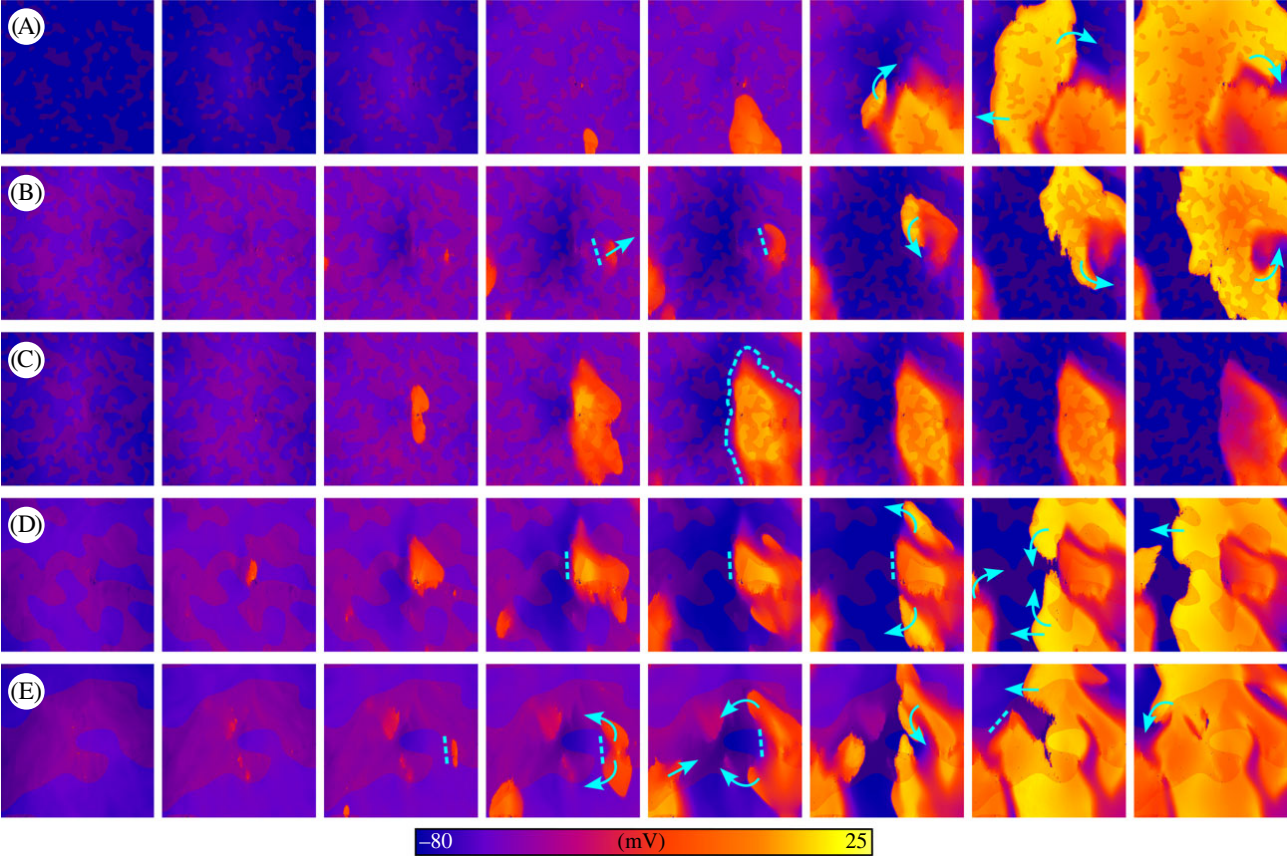
Complex excitation patterns emerging from trigger–substrate interactions. (A–E) show different simulations in which complex focal excitation patterns were observed, driven by interactions between DADs and TA. Blue arrows indicate wavefront propagation, dotted lines indicate regions of temporary conduction block and fibrosis patches are shown in semi-transparent dark red for context and relation of spatial properties. Results shown are for the mCRN cell model in control conditions with ISO, and a pre-pacing cycle length of 400 ms.

In a subset of simulations, complex excitation patterns emerged comprising multiple foci and DAD regions that interact to cause conduction block and re-entrant-like excitation patterns (figure 11, electronic supplementary material, video S5). This behaviour was only observed using the mCRN model, and simulations visualized in figure 11 were all using the control cell model with ISO implemented (see Discussion—Non-refractory mechanisms for unidirectional conduction for further discussion). These conditions were not sufficient, in our simulations, to lead to sustained re-entry on their own. However, the inclusion of fibroblast coupling did provide the substrate to both generate and sustain re-entry.

**Figure 12.**
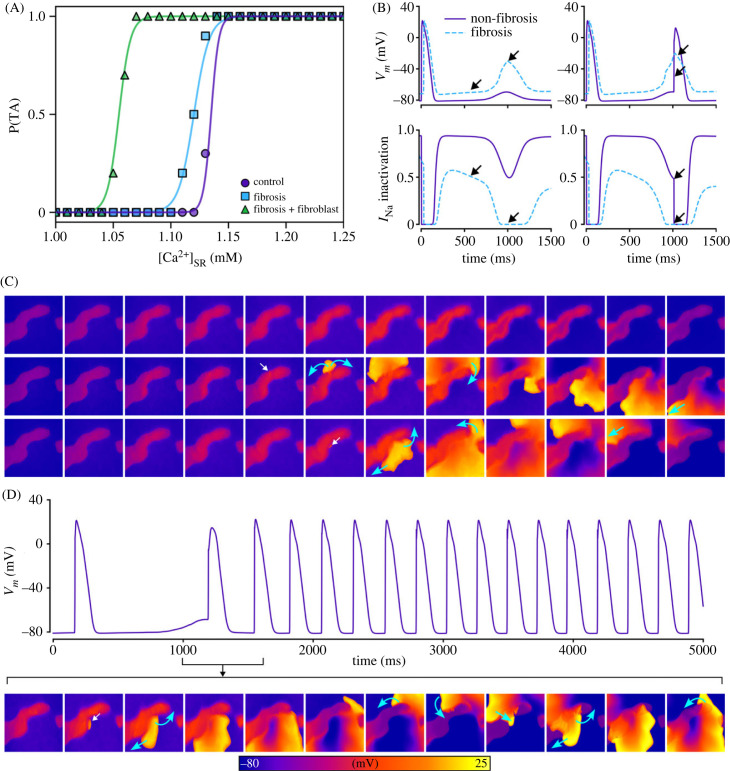
Impact of fibroblast coupling: unidirectional conduction patterns and spontaneous re-entry. (A) Impact of the inclusion of fibroblast coupling on the vulnerability to focal excitations. (B) Illustration of the mechanism by which inactivation of *I*_Na_ prevents TA in fibrosis regions. (C) Examples of unidirectional activation patterns that emerge from fibrosis regions when fibroblast coupling was included. (D) Example of spontaneously initiated sustained re-entry that emerges as a result of this unidirectional conduction. White arrows indicate regions of emergence, blue arrows indicate wavefront propagation and fibrosis patches are shown in semi-transparent dark red for context and relation of spatial properties. Results shown are for the minimal cell model with AF remodelling and ISO, and a pre-pacing cycle length of 400 ms.

**Figure 13.**
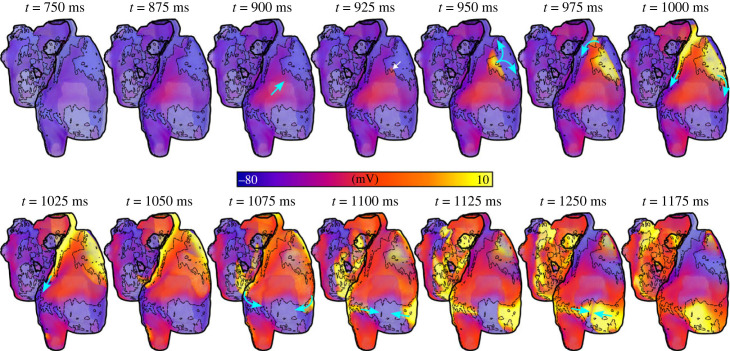
Complex three-dimensional excitation pattern occurring with fibroblast coupling. Temporal snapshots of membrane potential in the three-dimensional atria (heart 1) for a simulation implementing fibroblast coupling in the fibrosis regions. Excitation emerges unidirectionally (*t* = 925 ms) and propagates in a re-entrant like pattern around the fibrosis region. The white arrow indicates the point at which excitation emerges into the non-fibrotic atria, and the blue arrows indicate wavefront propagation. This simulation was performed with the minimal model, implementing AF remodelling and fibroblast coupling, with *σ*_ti_ = 0.1 and *σ_λ_* = 0.5.

The positive shift in resting membrane potential as a result of fibroblast coupling (figure 1) promoted focal excitations compared to both the control and fibrosis without fibroblast coupling models. This is best illustrated in the left-ward shift of the dependence of triggered activity on [Ca^2+^]_SR_ (figure 12A). Moreover, the inactivation of *I*_Na_ associated with the resting membrane potential depolarization (figure 12Ba,c) enabled large DADs that did not manifest as full excitation in the fibrosis region. These DADs could, however, excite the normal working myocardium at the border zone, emerging as a unidirectional wavefront (figure 12Bb,c). Under the right conditions, this unidirectional wavefront could re-enter into the fibrosis region and lead to sustained re-entrant excitation (figure 12C, electronic supplementary material, video S6). This mechanism is confirmed in the three-dimensional models (figure 13 and electronic supplementary material, video S7) although a full re-entrant circuit was not observed in any conditions that we tested.

**Figure 14.**
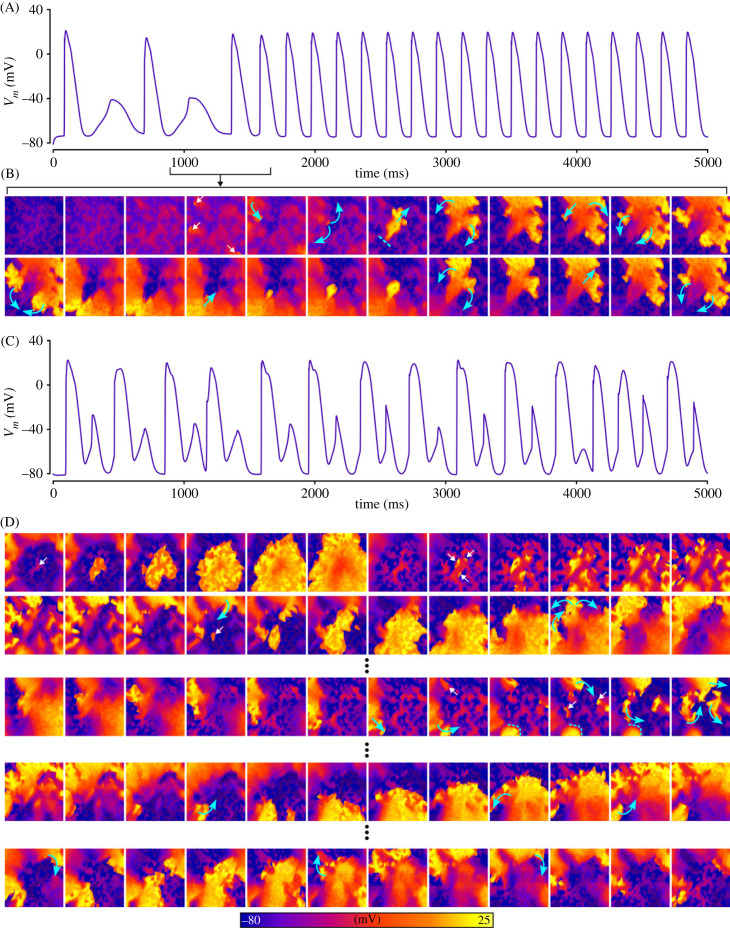
Spontaneous generation of arrhythmia. Two examples are shown of spontaneous arrhythmia generation that is driven by complex trigger–substrate interactions. (A) A simulation in which a stable re-entrant pattern emerges. (B) A simulation in which re-entry and focal excitations continuously interact. White arrows indicate regions of large cellular TA that may or may not fully induce a focal excitation, blue arrows indicate wavefront propagation, dotted lines indicate regions of temporary conduction block, and fibrosis patches are shown in semi-transparent dark red for context and relation of spatial properties. Results shown are for the minimal cell model with AF remodelling and ISO, and a pre-pacing cycle length of 400 ms.

When SCRE parameters were set to be rapid (early initiation time with tight synchronization), complex excitation patterns and spontaneous arrhythmia readily emerged in the presence of fibrosis and fibroblast coupling (figure 14A). Multiple focal excitations interacted with each other to promote substantial breakup of the wavefront that could degenerate into re-entry. If sufficiently rapid, focal excitations could occur during the re-entrant timeframe (figure 14B) leading to a complex pattern of wave breakup, emergence of re-entrant circuits, wave collision and termination of re-entrant circuits and regeneration of focal activity, that all interplay to drive complex fibrillatory dynamics (best appreciated by viewing electronic supplementary material, video S8). During this time, different quasi-stable re-entrant patterns emerged, before being interrupted by focal excitations, with the global pattern continuing to evolve.

## 4. Discussion

### 4.1. Summary of major findings

In this study, we applied novel computational models to investigate the impact of fibrosis on arrhythmia mechanisms. We focused on the role of fibrosis in promoting focal excitations and the interactions of those triggers with the arrhythmia substrate. Through this we have demonstrated that:

1. Patchy fibrosis led to an overall slowing down of activation time and increased heterogeneity in activation wavefronts.
2. The heterogeneously slowed conduction resulted in wavebreaks, providing a substrate for transient or sustained re-entry, with clear correlations between wavebreak locations and fibrosis patches.
3. Patchy fibrosis promoted the emergence of focal excitations by reducing electrotonic load but enabling propagation of initiated electrical waves; focal excitations were preferentially localized to fibrotic patches.
4. Both synchronization and magnitude constraints were substantially reduced by fibrosis.
5. Patch size was less important than total fibrosis area, implicating that relatively small fibrotic patches are sufficient to promote focal excitations.
6. Fibrosis also led to low probability events that had the potential to irregularly interrupt sinus rhythm.
7. Fibrosis promoted complex trigger–substrate interactions that could readily result in non-refractory mediated unidirectional conduction patterns.
8. The presence of myocyte–fibroblast coupling provided the most pro-arrhythmogenic condition, wherein large subthreshold DADs could unidirectionally emerge into healthy tissue and spontaneously generate transient or sustained arrhythmia.

### 4.2. Model of fibrosis

Many modelling and experimental studies have demonstrated that fibrosis results in increased conduction anisotropy and the introduction of substantial transverse conduction delay, with longitudinal conduction largely unchanged [1,16,17,36]. Increased collagen deposits preferentially occur between myocytes in the lateral direction, resulting in a collection of largely separated myofibre bundles [24,25]. These features were incorporated into our model through the random removal of 80% of transverse and 20% of longitudinal inter-nodal connections. The choice of these parameters resulted in only small changes to the activation time and average conduction velocity in the longitudinal direction, with much more substantial increase in the activation times in the transverse direction. Whereas this depended on the specific fibrosis structure, as well as the total tissue area fibrosis occupied, these features are comparable to the reduction in transverse conduction velocity observed in experiment. For example, de Bakker *et al*. [36] observed that conduction velocity in the perpendicular direction to myocyte orientation could be less than 10% that observed longitudinally; our results were closer to 20% than 10%, ensuring that our model does not represent an extreme of the condition. Whereas we estimated specific parameter values of 80% and 20% removal, we consider that replicating these critical features, pertaining to the significant reduction in transverse conduction, minor reduction to longitudinal conduction and an underlying structure of loosely connected one-dimensional strands, as a valid and valuable model for studying the effects of fibrosis on electrical excitation.

### 4.3. Mechanism of DAD-mediated focal triggers in fibrotic tissue

The precise mechanisms by which independent cellular SCRE can induce DADs that are sufficient to overcome the source–sink mismatch and manifest as a focal excitation in tissue have long been debated [18]. Simulation studies have recently begun to address these questions, highlighting mechanisms that enable the manifestation of these independent cellular events in whole-organ [21,38]. In our previous study, we demonstrated that electrotonic interactions play a dual role in supressing TA but also propagating DADs, which enables later timed SCRE to more easily overcome this mismatch [23]. However, in these simulations, a large volume of tissue undergoing relatively synchronized and large magnitude SCRE was generally required to induce a tissue-wide event.

In the present study, supporting previous results [18,20,39], we have demonstrated that fibrosis reduces the constraints required for a focal excitation. Xie *et al*. [18] showed that a loss of cellular connections substantially reduced the number of cells undergoing a DAD required for a focal excitation (from thousands to hundreds). They posited the explanation that fibrosis shifts the underlying tissue substrate from well-connected three-dimensional tissue to loosely connected one-dimensional strands, reducing the electrotonic load and enabling the overcoming of the source–sink mismatch. However, this study was conducted in the context of simultaneous, deterministic DADs that shared magnitude and timing, with the focus being on the number of cells undergoing these dynamics that are required to manifest in tissue. Campos *et al*. [20] included stochastic variability (i.e. cellular independence) of DADs in their study, in the context of an idealized isthmus within a scar region, demonstrating this provided the conditions for DAD-mediated conduction block and re-entry.

We have applied stochastic variability in the context of multiple fibrosis structures, with a microscopically detailed model of fibrosis. This approach enabled us to explore the impact of fibrosis distribution and patch size in combination with considerations on the variation of both timing and magnitude of SCRE. Our results, therefore, reveal the electrical patterns that underlie the emergence of a focal excitation driven by *independent* cellular events.

Our results demonstrated that a larger total proportion of fibrotic tissue promoted the emergence of focal excitations but that the size of the patches themselves had very small impact. The first result is easily explained: the constraints on supra-threshold dynamics in fibrotic tissue are less than in control tissue, and a larger area of fibrotic tissue increases the probability that supra-threshold SCRE dynamics occur within a fibrotic region. This also enhanced the probabilistic nature of induced focal excitations, leading to parameter regions where low probability TA was observed, compared to the sharp transition from 0% to 100% probability that is observed in control.

The observation that patch size had very little impact on the vulnerability to focal excitations implies that the smallest patch size we implemented (average size of approximately 44 mm^2^ for patch seed 10 in the 25% total area model) provided sufficient space for a focal excitation to emerge. Note that this is not the same statement that this area of tissue (approximately 49 nodes) was sufficient to induce a focal excitation on its own, as these areas of tissue are still influenced by DADs propagating from healthy regions that help raise the resting potential and bring the tissue region closer to the threshold for full excitation, even before any localized SCRE occurs. However, it does highlight how substantially the constraints are reduced by fibrotic tissue. Future studies could investigate even smaller patches, although we argue that this begins to transition into globally diffuse or interstitial fibrosis as opposed to the patchy fibrosis that is the focus of this manuscript.

We demonstrated that these results translate across both synchronization and magnitude variation: large magnitude and unsynchronized events, or small magnitude and highly synchronized events, were both sufficient to generate focal excitations in fibrotic regions.

### 4.4. Differential loss of transverse and longitudinal connections is a relevant controller of arrhythmia dynamics

In the present study, it was demonstrated that preferential loss of transverse connections [1,24,25,36] helped promote focal excitations (previous section) by providing both robust coupling within one-dimensional strands and reduced electrotonic load between strands. This supports the original study of Xie *et al*. [18] that found the loss of transverse connections specifically to be the most arrhythmogenic. This heterogeneity also promoted functional conduction block during rapid pacing, which contributed to the generation of re-entry and played an important role in complex excitation emerging from trigger–substrate interactions. The importance of reduced transverse coupling has been demonstrated in a previous study of the co-authors [40], where it was shown that these connections determined transverse conduction delay and affected the importance of both *I*_Na_ and *I*_CaL_ to successful propagation. Together with other previous implementations of highly heterogeneous conduction media associated with fibrosis (e.g. [11,41]), these studies collectively highlight the importance of capturing this underlying and complex coupling heterogeneity in computational models of fibrosis, as these features are not well reproduced in simpler models of fibrosis that rely on reducing the diffusion coefficient and/or increasing the anisotropy ratio.

### 4.5. Non-refractory mechanisms for unidirectional conduction and re-entry

Previous studies have highlighted novel mechanisms by which SCRE and DADs may promote the development of arrhythmia, beyond the established mechanisms of focal excitations that interact with cellular refractoriness, leading to unidirectional conduction block and re-entry. Liu *et al*. [21] demonstrated that DADs in one region of tissue could inactivate *I*_Na_ and block the excitation wavefront emerging from another region of tissue, leading to both unidirectional conduction patterns and re-entry. In Campos *et al*. [42], it was demonstrated that subthreshold DADs in the isthmus within a scar could provide the necessary conditions for both conduction block of the incoming wavefront and the degeneration into re-entry.

This present study contributes to the growing understanding of these mechanisms by elucidating how triggers and substrates can interact to form complex excitation patterns. Similar to Liu *et al*. [21], we observed conditions wherein DADs and TA simultaneously exist and interact to lead to complex and re-entrant-like excitation patterns; these conditions were substantially promoted by the presence of fibrosis. In particular, the heterogeneity induced by patchy fibrosis promoted localized regions of TA-inducing DADs (within fibrotic patches) and non-TA-inducing DADs (in non-fibrotic regions). These more complex dynamics were observed primarily using the mCRN model and not in the minimal model. This was attributed to the differing resistance to a DAD in each model. The minimal model had less opposing current and subsequently full TA was induced with lower magnitude SCRE than in the mCRN model. It was these larger magnitude but subthreshold SCRE that were required to sufficiently induce *I*_Na_ inactivation and result in conduction block; in the minimal model, there was no parameter region that we could find where substantial conduction block and focal excitations simultaneously occurred. Such results highlight one limitation of using a minimal model that does not contain all of the relevant components that act in the resting potential region. However, this was the only result that differed qualitatively between the minimal and mCRN models.

Additionally, the introduction of fibroblast coupling (which, in this present study, was implemented simply as a positive shift in the resting membrane potential) provided a substrate that readily facilitated unidirectional and re-entrant-like conduction patterns. The elevation of resting membrane potential inactivated *I*_Na_, enabling much larger DADs that do not trigger full activation of *I*_Na_ and therefore do not lead to cellular TA. These DADs could propagate asymmetrically in the fibrotic region and, upon reaching the border zone with the surrounding healthy tissue, they could emerge into this tissue as a full excitation. This often occurred unidirectionally, as the excitation wave was frequently only initiated at one location of the border zone and could not initially enter the fibrotic region due to *I*_Na_ inactivation. The wave would then propagate throughout the remainder of the tissue, travelling along the border zone of the fibrotic region, until it was able to enter this region fully if it had sufficiently recovered within this timeframe. This provided the most vulnerable substrate for the spontaneous development of re-entry.

Notably, this novel mechanism, which relies on heterogeneously inactivated *I*_Na_, requires less extreme SCRE than a rapidly timed ‘S2’ stimulus occurring in a small spatio-temporal window, as is required for the more established mechanisms of conduction block. As a result, we speculate that it is possible that these mechanisms may be more common in the calcium-induced, DAD-mediated spontaneous generation of arrhythmias compared to the traditional refractory-based conduction block mechanisms. Novel treatment strategies that aim specifically to inhibit this mechanism may lead to more effective options than those currently available, in particular, in patient populations for whom the current treatment strategies are not efficacious. The validity and relevance of these novel mechanisms requires confirmation in both experiment and the clinic before these ideas are pursued further. We note that a recent study found that myocyte–fibroblast coupling in scar regions to be highly arrhythmogenic [32] and it *may* be that the mechanism described above is relevant for these observations.

## 5. Limitations

Relatively large tissue sizes were implemented in the two-dimensional simulations of this study that may be larger in total surface area than the healthy human atria. This choice was made to provide sufficient space for sustained re-entrant excitation and for complex fibrosis patterns to be imposed. We note that the total activation time in the two-dimensional tissue models (approximately 110 ms) was similar to the three-dimensional anatomically detailed models and that expected in human atria. Whereas this is not a direct comparison (two-dimensional models were paced from the centre of the tissue, whereas the pacemaker of the atria, the SAN, is not centrally placed), it confirms that the excitation wavelength is approximately the correct size in relation to the total tissue area. Moreover, many conditions and diseases in which AF occurs are associated with an enlargement of the atria (e.g. due to hypertrophy or dilatation) beyond the total surface area implemented in our two-dimensional simulations.

It is also worth clarifying the nature of the fibre orientation models implemented in two dimensions. These models were not rigorously developed or based on detailed data describing remodelling. Rather, the intention of these three models was to identify if the organization of fibre orientation had an impact on either trigger or substrate dynamics. For this purpose, the control varying field was to ensure a more realistic context than the perfectly idealized case (the importance of which can be seen in the activation patterns) and the remodelled field was to introduce areas of abrupt changes in fibre angle that were not present in the control model. It would take substantially more work, beyond the scope of the aims of this study, to develop well-validated fibre structure models, and it would then also be prudent to include multiple models of fibre structure to avoid any model-specific conclusions. This would have added substantial simulation time to the already intensive requirements of this study. However, we note that the fully idealized condition is a common way of performing two-dimensional simulations (e.g. [16]), and therefore we believe that introducing some variation in fibre structure, even if it has not been rigorously validated, still offers more realism than relying on such an idealized case.

Currently, our models lack the implementation of regional differences in electrophysiological properties that have been included in numerous previous studies (e.g. [43,44]). Moreover, the fibrosis model used in our study is still relatively simple (random removal of a set proportion of axial and transverse connections within fibrosis regions). A more detailed model which incorporates data-informed connection maps at the cellular scale may offer more insights. Previous studies have indicated that intramural conduction in the atria could be an important regulator of arrhythmia dynamics, particularly in the presence of fibrosis (e.g. [13,45,46]). These intramural properties were not accounted for in the present model, but could be particularly relevant for focal excitations and the interaction between focal excitations and re-entry.

In addition to fibrosis, connexin remodelling and the dynamics of gap junctions may also play significant roles in arrhythmia development [24,25,47,48]. For example, lateralization of connexins could somewhat counteract the loss of transverse coupling introduced by increased collagen between myofibre bundles, although it is likely these concomitant changes still result in increased anisotropy in the remodelled atria. Considering these factors in future models could offer further insights into the mechanisms underlying arrhythmia induction and progression. While our fibroblast model reproduced the impact of resting potential homogeneously within fibrotic regions, a more detailed model considering the actual coupling of a varied number of fibroblasts to cardiomyocytes may introduce additional heterogeneities, providing a more accurate depiction of fibrosis-induced effects on cardiac electrophysiology.

Our results where focal excitations interacted with, and sometimes suppressed sinus rhythm, were performed in the context of applied stimuli. The natural pacemaker potentials of the SAN were not included in the present model. This would be a necessary inclusion to further understand the interaction of focal beats and sinus rhythm. For example, if a focal beat were received by the SAN, inducing an AP in this region, then this would delay the next sinus beat in a way that was not captured by the predefined stimulus times implemented in the present study. Such interactions may offer valuable insights into the generation of arrhythmia.

Additionally, the current computational simulations have limitations in terms of efficiency, particularly for full three-dimensional simulations over extended time periods (more than 10 s of simulation time). In combination with the requirement for a high volume of simulations necessary to characterize the statistics of these probabilistic events as well as ensure all behaviour is captured, this presents numerous challenges for feasible delivery of simulation results in a reasonable timeframe. Developing a more efficient method for conducting full three-dimensional simulations would be highly valuable and would enable more complex, long-term behaviours to be considered.

## 6. Conclusion

In this study, we have demonstrated that patchy fibrosis promotes arrhythmia triggers, substrate and trigger–substrate interactions that can both generate and maintain AF. In particular, a loss of cellular connections facilitates the emergence of focal excitations (arrhythmia triggers) while also promoting complex excitation patterns that can lead to wavebreak and re-entry (substrate). The simultaneous presence of both features enabled subthreshold release events and focal excitations to coexist and interact to develop into re-entry without the requirement for refractory-based conduction block. Fibroblast coupling enhanced these interactions by enabling localized large subthreshold release events that can emerge unidirectionally into the surrounding atria. This study has, therefore, revealed the mechanisms by which fibrosis promotes arrhythmia triggers while also revealing novel mechanisms of arrhythmia induction that could be important for clinical management of the condition.

## Data Availability

Model code, including simulation geometries and fibrosis maps, can be found on Zenodo: Colman, M. A. (2023). Multi-scale Cardiac Simulation Framework (Version 1.4). Michael Colman https://doi.org/10.5281/zenodo.10204625 [49]. Supplementary material is available online [50].
